# Deep muscle stimulator in the treatment of post-stroke spasticity: A protocol for systematic review and meta-analysis

**DOI:** 10.1097/MD.0000000000033602

**Published:** 2023-04-28

**Authors:** Junfang Lei, Chengdong Zhang, Jialin Gai, Xiaohua Fan, Jiqin Tang

**Affiliations:** a College of Rehabilitation Medicine, Shandong University of Traditional Chinese Medicine, Jinan, China; b Rehabilitation Medicine, Weifang Medical University, Weifang, China; c Rehabilitation Department, Shandong Provincial Hospital, Jinan, China; d Hospital Development Planning Division, Shandong University of Traditional Chinese Medicine, Jinan, China.

**Keywords:** deep muscle stimulator, network meta-analysis, post-stroke spasticity, protocol, systematic review

## Abstract

**Background:**

Spasticity is one of the most common complications and sequelae of stroke, with the main clinical manifestations being increased muscle tension, pain, stiffness, and other disorders. It not only increases the length of hospitalization and medical costs but also affects the quality of daily life and the stress of returning to society, increasing the burden on patients and their families. At present, 2 driver types of deep muscle stimulator (DMS) have been used in the clinical treatment of post-stroke spasticity (PSS) with good clinical results, but there is no evidence of clinical efficacy and safety. Therefore, this study aims to integrate direct and indirect comparative clinical evidence through a systematic review and network meta-analysis (NMA). According to the data, different driver types for DMS with the same body of evidence will be collected, analyzed, and sequenced in a quantitative and comprehensive manner and then screened for the optimal driver type of DMS device for PSS treatment. The study also aims to provide reference value and an evidence-based theoretical basis for the clinical optimization of DMS equipment selection.

**Methods:**

A comprehensive retrieval of China National Knowledge Infrastructure, Chinese scientific journal database, China biological feature database, Wanfang Chinese databases and the Cochrane Library, PubMed, Web of Science, and Embase foreign databases will be conducted. Randomized controlled trials of these 2 driver types of DMS devices combined with conventional rehabilitation training of PSS will be searched and published. The retrieval time is from the establishment of the database to December 20, 2022. The 2 first authors will screen references that meet the inclusion criteria, independently extract data according to predesigned rules, and assess the quality of the included studies and the risk of bias according to the Cochrane 5.1 Handbook criteria. R programming and Aggregate Data Drug Information System software will be used to perform a combined NMA of the data and to evaluate the probability of ranking for all interventions.

**Results:**

The NMA and probability ranking will determine the best driver type of DMS device for PSS.

**Conclusion:**

This study will offer a comprehensive evidence-based approach to DMS therapy and assist doctors, PSS patients, and decision-makers in selecting a more efficient, secure, and cost-effective treatment option.

## 1. Introduction

Cerebral stroke is a disease with high morbidity, high mortality, and high disability. It has been reported that the incidence of stroke patients and the number of years of disability adjustment have decreased in China in recent years, but it is still higher than in developed countries and is the first major disease causing death and disability in Chinese adults.^[1,2]^ With rapid social and economic development, an accelerated pace of life, and increased work pressure, the incidence of this disease is on the rise and tends to be among younger people, which seriously disturbs the normal life and work of patients but also imposes a heavy burden on families and society. In China, there are approximately 2 million new stroke patients every year, and most of them need family care due to complications.^[3,4]^ Post-stroke spasticity (PSS) is one of the common complications and sequelae of stroke, with a prevalence ranging from 19 to 40% in different studies, but mainly occurs in the late mid-stroke period.^[5,6]^ Spasticity is a type of upper motor neuron injury syndrome characterized by increased muscle resistance caused by external draft, which is speed- and muscle-length dependent.^[7]^ Currently, the main treatments for PSS include physical therapy, occupational therapy, exercise therapy, hydrotherapy, physiotherapy, traditional rehabilitation therapy, oral drugs, neurolytic drug injection, and surgery, but there are some side effects or high treatment costs.^[8]^ In addition, a growing number of studies have shown that local vibration is an effective nondrug therapy in the treatment of PSS and has some advantages in improving motor function.^[9,10]^

Deep muscle stimulator (DMS) is a local vibration therapy that is painless, noninvasive, highly safe, and well tolerated and can greatly improve the subjective initiative and treatment compliance of clinical patients. As a new green treatment, it has been used for the treatment of PSS and has achieved good rehabilitation outcomes.^[11]^ Therefore, in this study, 2 clinically common driving types of DMS will be selected for the treatment of PSS, namely, mechanically driven DMS and air-driven DMS. Conventional rehabilitation therapy or sham muscle vibration stimulation will be selected for the control group. The clinical efficacy of local vibration for the treatment of PSS has been compared, but the efficacy and safety of the 2 different driving types of DMS for the treatment of PSS have not been evaluated evidence-based. Therefore, the purpose of this study is to use the network meta-analysis (NMA) method to integrate the clinical evidence of direct and indirect comparative relationships. Different driver types of DMS with the same body of evidence for the treatment of PSS will be collected, analyzed, and ranked in a quantitative and comprehensive manner, and then the advantages and disadvantages of the efficacy and safety of different driving types of DMS will be discussed to obtain the optimal treatment plan, to provide reference value and evidence-based medical evidence for clinical optimization of device selection.^[12]^

## 2. Materials and Methods

### 2.1. Protocol and registration

We will complete this systematic evaluation and NMA protocol in accordance with recognized standards, which follow the statements of the Cochrane manual standard, the Grading of Recommendations Assessment, Development, and Evaluation (GRADE), and “protocol for systematic evaluation and network meta-analysis”. A report on the further results of this study will be submitted in accordance with the guidelines of the PRISMA NMA extension statement. We obtained the registration number (CRD42023409153) of this study on the PROSPERO platform (https://www.crd.york.ac.uk/prospero/). It does not involve private information and does not require further ethical approval and informed consent.

### 2.2. Information sources

A literature search will be conducted for clinical randomized controlled studies of 2 different driver types of DMS for PSS using computerized search techniques. The primary search will be selected, setting the time from database creation to December 20, 2022. The computer retrieval electronic database included the China National Knowledge Infrastructure, China biological feature database, Wanfang data, Chinese scientific journal database, and other Chinese databases, as well as the Cochrane Library, PubMed, Web of Science and EMBASE, and other foreign databases. Chinese search terms included stroke, cerebral vascular accident, cerebral hemorrhage, subarachnoid hemorrhage, transient ischemic attack, cerebral thrombosis, cerebral embolism, spasm, spastic paralysis, increased muscular tension, PSS, local muscle vibration, DMS, regular rehabilitation training, random, etc. English search terms included (stroke OR cerebrovascular accident OR cerebral hemorrhage OR cerebral ischemia) and (spasm OR spastic paralysis OR increased muscular tension OR post stroke spasticity) and (vibration OR vibration stimulation OR vibration therapy OR vibration training OR Local muscle vibration OR focal muscle vibration OR deep muscle stimulator) and (regular rehabilitation training) and (Random OR randomized controlled trials OR clinical randomized controlled trials).

When searching the literature, subject terms and free terms should be searched separately, and a comprehensive search should be conducted using relevant free terms and terminology. Meanwhile, the research of the WHO international clinical trial registration platform and ClinicalTrials.gov will be searched to determine additional potential trial registrations. In addition, the relevant journals will be searched in the reference literature, and the relevant literature will be tracked. Google Scholar (https://scholar.google.com)should be used in conjunction with Baidu Scholar (https://xueshu.baidu.com) and other relevant search engines, and data will be provided for all relevant authors and principal investigators to supplement incomplete reports of original papers or unpublished studies. Every effort will be made to ensure that initial search efforts are comprehensive so that valuable research material is not lost. At the same time, according to the participant-intervention-comparator-outcomes-study design search principle, we will include research that meets the standards and organize and create the database. In Table 1, the preliminary search strategy of the PubMed database is taken as an example to summarize the preliminary search strategy that will be adapted to the requirements of other keyword-related electronic databases.

**Table 1:** Search strategy used in PubMed database.

Number	Search terms
#1	Search “Stroke” [Mesh]
#2	Search ((cerebrovascular accident [Title/Abstract]) OR (cerebral hemorrhage [Title/Abstract])) OR (cerebral ischemia [Title/Abstract])
#3	#1 OR #2
#4	Search “Spasm” [Mesh]
#5	Search ((spastic paralysis [Title/Abstract]) OR (increased muscular tension [Title/Abstract])) OR (post stroke spasticity [Title/Abstract])
#6	#4 OR #5
#7	Search “Vibration” [Mesh]
#8	Search (((((vibration stimulation [Title/Abstract]) OR (vibration therapy [Title/Abstract])) OR (vibration training [Title/Abstract])) OR (Local muscle vibration [Title/Abstract])) OR (focal muscle vibration [Title/Abstract])) OR (deep muscle stimulation [Title/Abstract])
#9	#7 OR #8
#10	Search (regular rehabilitation training [Title/Abstract])
#11	Search “Randomized Controlled Trial” [Publication Type]
#12	Search (((((Controlled Clinical Trial [Title/Abstract]) OR Randomized [Title/Abstract]) OR Placebo [Title/Abstract]) OR Randomly [Title/Abstract]) OR Trial [Title/Abstract]) OR Groups [Title/Abstract]
#13	#11 OR #12
#14	#3 AND #6 AND #9 AND #10 AND #13

This search strategy will be modified as required for other electronic databases.

### 2.3. Eligibility criteria

The design of the inclusion criteria and exclusion criteria in this study is based on the 5 main principles of the participant-intervention-comparator-outcomes-study.

#### 2.3.1. Type of participant.

The patient is a single PSS with no restrictions on gender, race, or region. The diagnostic criteria for PSS should conform to the following requirements: stroke patients with definite diagnostic criteria, confirmed by computed tomography or magnetic resonance imaging, accompanied by 1 limb spasm or muscular hypertonia, and tendon reflex.

#### 2.3.2. Type of interventions and comparators.

When there are clear diagnostic criteria, efficacy judgment criteria and basic treatment agreement, mechanical vibration DMS or air vibration DMS combined with routine rehabilitation training will be used as the intervention measure for the experimental group. In the control group, routine rehabilitation training or sham muscle vibration stimulation is selected as the intervention measure.

#### 2.3.3. Type of outcomes.

The predetermined evaluation results mainly include spasticity, motor function, and clinical efficacy. The evaluation of spasticity mainly includes the modified Ashworth scale (MAS), and the clinical spasticity index (CSI). The evaluation of motor function mainly includes the Fugl-Meyer Assessment (FMA), activity of daily living (ADL), function independence measure (FIM), Barthel index (BI), or modified BI (MBI).

#### 2.3.4. Type of study.

The literature included randomized controlled trials (RCTs) with no limitations on language and blinding or assignment concealment. Cluster RCTs will be included when cluster effects are considered. Although an increasing number of clinical trials are reported from mainland China, there are concerns about the quality of these studies, so it is specifically stated that we will include Chinese trials as long as they are approved by the local institutional review committee and registered in the international database. In addition, the author will exclude non-RCTs, case reports, experience summaries, self-control and review literature, animal experimental research, and repeatedly published literature. Meanwhile, studies with an unclear diagnosis of PSS or literature combining with other diseases, unclear criteria for judging the efficacy of the trial and control groups, treatment measures involving other treatments, literature affecting causality judgments, literature with unclear study results of the final treatment, incomplete data or no connection to the full-text authors will be also excluded.

### 2.4. Study selection and data extraction

**Figure 1.**
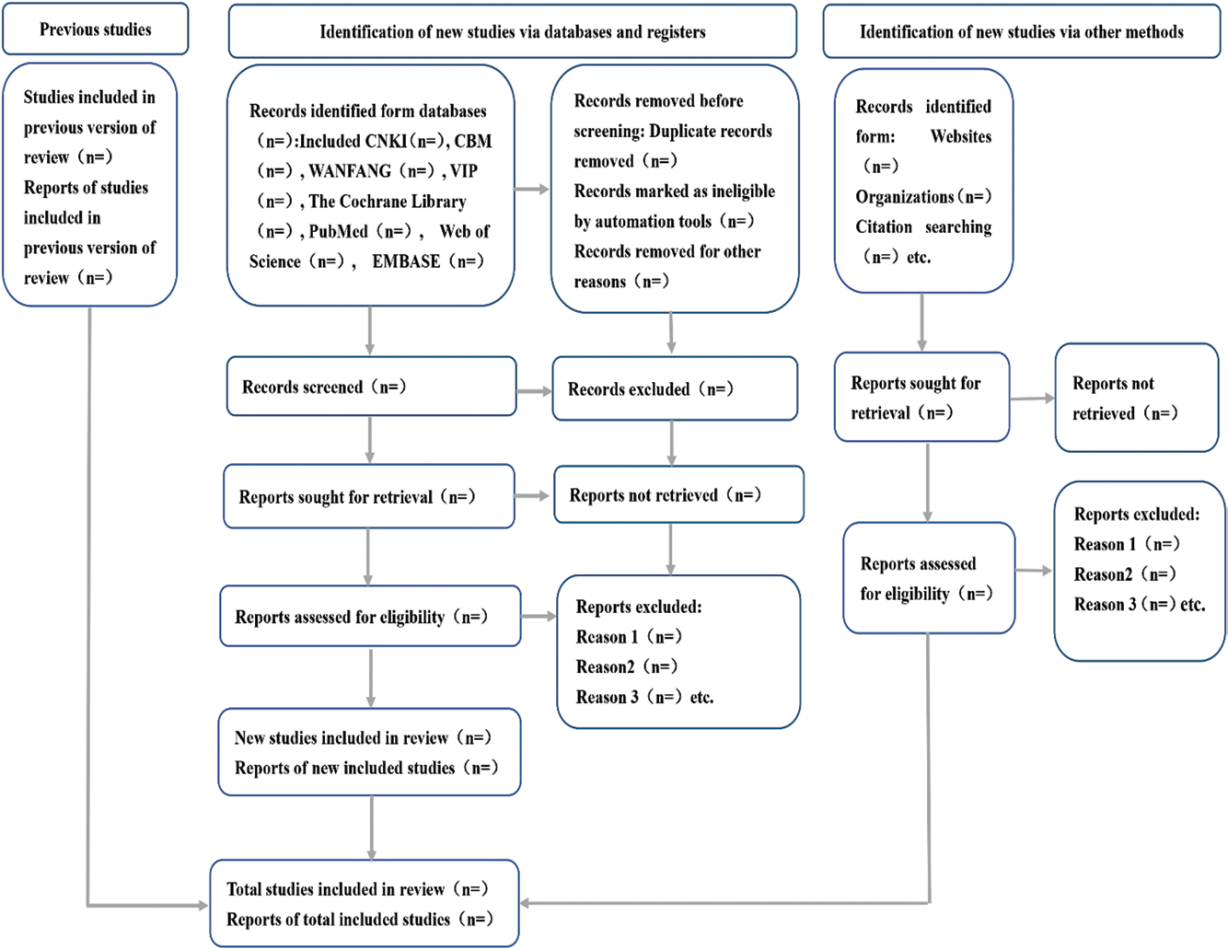
Flow diagram of study. CBM = China biological feature database, CNKI = China National Knowledge Infrastructure. VIP = Chinese scientific journal database.

According to the retrieval strategy of the above electronic databases, 2 researchers searched the electronic databases in Chinese and English, used Endnote X9 software (https://endnote.com) to search for duplicate information, combined the literature retrieval results in different databases, established the information database, and downloaded the full text. Then, 2 first authors independently extracted the data for preliminary screening, extracted the data according to the predetermined table, cross-checked and reviewed, marked the reasons for each excluded study, invited a third review researcher to jointly discuss, and made a final decision on the research with different opinions. The process of study selection is summarized in the PRISMA flowchart in Figure 1.^[13]^

### 2.5. Data extraction and management

Data extraction content includes

1. Basic information of the included literature (including the first author, published journal and year, and research topic).
2. Relevant information about the treatment group and the control group in the literature (including the number of cases, total cases, age, intervention measures, frequency and amplitude and time of vibration, course of treatment, and outcome indicators).
3. Design type and quality evaluation information of the included literature.
4. Outcomes (The outcome indexes include clinical efficacy, MAS, CSI, FMA, ADL, FIM, and BI or MBI score).

### 2.6. Study quality evaluation

According to the quality evaluation standard of the Cochrane (https://www.cochrane.org) system evaluator manual, the Review Manager quality evaluation tool (https://training.cochrane.org/online-learning/core-software/revman) will be used to evaluate the methodological quality of the included studies, including the random method, assignment concealment, blinding method, outcome data integrity, selective reporting, number of dropped cases, follow-up and other biases. Each project is divided into 3 options: high risk, low risk, and uncertainty risk, according to the description of the above aspects in the included research. The 2 first authors independently completed the quality evaluation results of the included literature. In case of discrepancies in the results, it is necessary to invite a third researcher to help each other with interpretation and quality assessment according to the standards of the Cochrane manual, literature quality assessment, and bias risk assessment. Statistical software, data integration, and NMA will be carried out by R language programming software (https://www.r-project.org).^[14,15]^

Referring to the GRADE handbook, the assessment performed by 2 independent authors through the GRADE (https://gradepro.org/), will be designated into 4 grades: high quality, moderate quality, low quality, and very low quality.^[16]^

### 2.7. Data synthesis and statistical methods

#### 2.7.1. Pairwise and NMA.

Review Manager software will be used for the assessments of literature quality and risk of bias. Aggregate Data Drug Information System (ADDIS, http://drugis.org/addis) and R software will be employed for direct and indirect result comparisons and 95% confidence interval calculations in the NMA. Meanwhile, anecdotal sequence and network relationship diagrams of the 2 driver types DMS will be constructed to reveal the indirect comparative relationships between them. The node indicates a DMS, the line denotes a direct or indirect comparative relationship between 2 types of DMS, and the line thickness reflects the number of included RCTs. Subsequently, all direct and indirect comparisons will be evaluated to determine the most effective DMS for PSS among these 2 kinds of DMS and estimate the rank probability of DMS using the Markov chain Monte Carlo (MCMC) method. The “bmeta” program in the R package will be used, and the Bayes MCMC algorithm will be called to analyze the random effects model results.^[17,18]^

The odds ratio and 95% confidence interval will be utilized for efficacy analysis. All measurement data are presented as standardized or weighted mean differences. According to the NMA probability ranking, the higher the clinical efficacy, FMA, ADL, FIM, and BI or MBI score (Rank 1), the greater the effects, while the smaller the MAS and CSI score, (Rank 1), the greater the effects. The data of the random effects model will be called by ADDIS software according to the Bayesian MCMC algorithm for prior evaluation and processing (4 chains are subjected to simulation modeling, and the initial value, iteration step, number of iterations, and number of simulation iterations are adjusted to 2.5, 10, 20,000, and 50,000, respectively).^[19]^

#### 2.7.2. Assessment of heterogeneity.

Heterogeneity will be evaluated by Cochrane. For each pairwise comparison, statistical heterogeneity will be evaluated by the *I*^2^ index, subgroup analysis based on the heterogeneity factors, and study by the *x*^2^ test. The clinical and methodological heterogeneity of the included studies will be assessed, and the fit of the fixed effects model and random effects model will be compared. If each study in the subgroup had statistical homogeneity (*P* ≥ .1, *I*^2^ ≤ 50%), the fixed effect model will be used for meta-analysis. Otherwise, the causes of heterogeneity will be analyzed first, the random effect mode will be used for meta-analysis without obvious clinical heterogeneity (*P* < .1, *I*^2^ > 50%), and the possible causes of heterogeneity will be identified in terms of clinical and methodological aspects. If the data provided for clinical trials are not amenable to meta-analysis, descriptive analysis should be performed.^[20,21]^

#### 2.7.3. Subgroup and sensitivity analyses.

If the result of the meta-analysis is positive and >3 studies are included, a sensitivity analysis of the statistical results will be performed using R software, the meta-analysis will be performed again for each excluded study, and the results will be compared with the results before exclusion. If there is no substantial change in the comparative analysis, the results are stable. If significant heterogeneity is found, subgroup analysis will be envisaged based on treatment time, age, race, sex, and quality of study to investigate possible sources of heterogeneity.^[21]^

#### 2.7.4. Assessment of inconsistency.

The Node-Split Model of ADDIS software will be used to test the inconsistency. If there is no significant difference (*P* > .05) in each study within the subgroup, it indicates that there is little heterogeneity in the included studies; therefore, the consistency model will be used for analysis. Otherwise, the inconsistency model will be used for analysis. The Potential Scale Reduced Factor reflects convergence. When Potential Scale Reduced Factor is close to 1 or equal to 1, it indicates that it has achieved better convergence efficiency and that the results of consistency model analysis are reliable.^[19]^

#### 2.7.5. Publication bias.

If >5 studies are included, R software will be used to analyze the potential publication bias, and the figure will be inverted funnel-shaped and symmetrical, which indicates a relatively low likelihood of publication bias. If the figures are biased, there is a greater possibility of publication bias.^[15]^

## 3. Discussion

DMS has the effect of relaxing contracture muscles and restoring muscle elasticity. In this study, mechanically driven DMS and air-driven DMS local muscle vibration stimulation instruments commonly used in clinical practice are used to induce proprioceptive input and Ia afferent fibers through frequency vibration to activate the primary end of the muscle spindle. It may lead to inhibition of the spinal reflex and thus inhibit the circuit of antagonistic muscle neurons, reduce the excitability of antagonistic muscle, and activate the active muscle, thereby improving spasm.^[22–24]^ The instrument has clinical application value because of its mobility, convenience, standardization, and safety, which greatly improve the clinical cure rate, shorten the course of the disease, and improve the safety and compliance of PSS treatment.

At present, there is no clear treatment standard for DMS in the treatment of PSS, and most studies only focus on the comparison of local muscle vibration therapy and systemic muscle vibration therapy, lacking NMA to compare the clinical efficacy and safety of different driving types of DMS in the treatment of PSS. Therefore, NMA will be used in this study to obtain outcome indicators such as clinical effects, spasmodic and motor function, and improvement scores of related symptoms of the 2 driving DMSs in the treatment of PSS, to clarify the therapeutic effects of the 2 driving DMSs. The research then ranks the probability according to the advantages and disadvantages of the index effect, to determine the best medical instruments and clinical treatment measures. The quality of this evidence will be reevaluated by the hierarchical method.

The protocol for the NMA has been registered on the international system review expectation register (CRD42023409153), which will follow the guidelines of the “Cochrane Intervention System Review Manual” and “PRISMA - P statement.” In addition, if changes to the protocol are needed, the reason and date of the change will be stated.

## Author contributions

**Conceptualization:** Junfang Lei, Chengdong Zhang.

**Data curation:** Junfang Lei, Chengdong Zhang, Jialin Gai.

**Formal analysis:** Junfang Lei, Chengdong Zhang, Jialin Gai.

**Funding acquisition:** Jiqin Tang.

**Investigation:** Junfang Lei, Chengdong Zhang, Jialin Gai.

**Methodology:** Junfang Lei, Chengdong Zhang, Jialin Gai, Jiqin Tang.

**Project administration:** Junfang Lei, Chengdong Zhang, Jialin Gai, Xiaohua Fan, Jiqin Tang.

**Resources:** Junfang Lei, Chengdong Zhang, Jialin Gai, Xiaohua Fan, Jiqin Tang.

**Software:** Junfang Lei, Chengdong Zhang, Jialin Gai, Jiqin Tang.

**Supervision:** Xiaohua Fan, Jiqin Tang.

**Validation:** Xiaohua Fan, Jiqin Tang.

**Visualization:** Junfang Lei, Chengdong Zhang, Jialin Gai.

**Writing – original draft:** Junfang Lei, Xiaohua Fan, Jiqin Tang.

**Writing – review & editing:** Junfang Lei, Xiaohua Fan, Jiqin Tang.
